# Association of vaginal dysbiosis and biofilm with contraceptive vaginal ring biomass in African women

**DOI:** 10.1371/journal.pone.0178324

**Published:** 2017-06-08

**Authors:** Liselotte Hardy, Vicky Jespers, Irith De Baetselier, Jozefien Buyze, Lambert Mwambarangwe, Viateur Musengamana, Janneke van de Wijgert, Tania Crucitti

**Affiliations:** 1 HIV and Sexual Health Unit, Department of Public Health, Institute of Tropical Medicine, Antwerp, Belgium; 2 HIV/STI Reference Laboratory, Department of Clinical Sciences, Institute of Tropical Medicine, Antwerp, Belgium; 3 Clinical Trials Unit, Department of Clinical Sciences, Institute of Tropical Medicine, Antwerp, Belgium; 4 Rinda Ubuzima, Kigali, Rwanda; 5 Institute of Infection and Global Health, University of Liverpool, Liverpool, United Kingdom; University of Pittsburgh, UNITED STATES

## Abstract

We investigated the presence, density and bacterial composition of contraceptive vaginal ring biomass and its association with the vaginal microbiome. Of 415 rings worn by 120 Rwandese women for three weeks, the biomass density was assessed with crystal violet and the bacterial composition of biomass eluates was assessed with quantitative polymerase chain reaction (qPCR). The biomass was visualised after fluorescence in situ hybridisation (FISH) and with scanning electron microscopy (SEM). The vaginal microbiome was assessed with Nugent scoring and vaginal biofilm was visualised after FISH. All vaginal rings were covered with biomass (mean optical density (OD) of 3.36; standard deviation (SD) 0.64). Lactobacilli were present on 93% of the rings, *Gardnerella vaginalis* on 57%, and *Atopobium vaginae* on 37%. The ring biomass density was associated with the concentration of *A*. *vaginae* (OD +0.03; 95% confidence interval (CI) 0.01–0.05 for one log increase; p = 0.002) and of *G*. *vaginalis* (OD +0.03; (95% CI 0.01–0.05; p = 0.013). The density also correlated with Nugent score: rings worn by women with a BV Nugent score (mean OD +0.26), and intermediate score (mean OD +0.09) had a denser biomass compared to rings worn by participants with a normal score (p = 0.002). Furthermore, presence of vaginal biofilm containing *G*. *vaginalis* (p = 0.001) and *A*. *vaginae* (p = 0.005) correlated with a denser ring biomass (mean OD +0.24 and +0.22 respectively). With SEM we observed either a loose network of elongated bacteria or a dense biofilm. We found a correlation between vaginal dysbiosis and the density and composition of the ring biomass, and further research is needed to determine if these relationships are causal. As multipurpose vaginal rings to prevent pregnancy, HIV, and other sexually transmitted diseases are being developed, the potential impact of ring biomass on the vaginal microbiota and the release of active pharmaceutical ingredients should be researched in depth.

## Introduction

Contraceptive vaginal rings are available in high income countries and Latin America but not in sub-Saharan Africa [1]. They are expected to be introduced there in the near future. Multi-purpose vaginal rings are being developed for the controlled release of drugs to prevent reproductive tract infections, such as HIV (dapivirine ring) [2], herpes simplex virus type 2 (HSV-2) [3], bacterial vaginosis (BV), and pregnancy [4].

Early contraceptive ring studies demonstrated that ring use did not negatively affect the naturally protective vaginal environment including the presence of lactobacilli [5–7]. Recent more in-depth work showed an increase in health-associated lactobacilli concentrations with ring use [8–10]. This effect was thought to be caused by ethinyl estradiol [8–10]. Lactobacilli are important in the two main states of the vaginal microbiome: the health-associated state is dominated by lactobacilli, and the BV-associated microbiome is characterised by polymicrobial dysbiosis. In dysbiosis, lactobacilli disappear and the concentrations of facultative anaerobic bacteria, such as *Gardnerella vaginalis* and *Atopobium vaginae*, increase [11]. These anaerobic bacteria will often form a vaginal biofilm [12–14]. Bacterial biofilms are also known to develop on indwelling medical devices [15]. The potential development of bacterial biofilm on vaginal rings in vivo has yet to be explored in humans.

We hypothesised that a biomass would develop on vaginal rings, and that rings worn by women with BV-associated dysbiosis would have higher biomass density than rings worn by women with dysbiosis. To investigate this hypothesis, we studied the presence, density and bacterial composition of the biomass on contraceptive vaginal rings and investigated the association between ring biomass density and the vaginal microbiota.

## Methods and materials

This is a laboratory sub-study of the “Ring Plus” contraceptive vaginal ring study performed at the Rinda Ubuzima (RU) research clinic in Kigali, Rwanda (ClinicalTrials.gov identifier NCT01796613) [16]. Participants were between 18 and 35 years old and provided written informed consent for participation in the study. The Ring Plus study was approved by the Rwanda National Ethics Committee, Rwanda (Approval number 481/RNEC/2013); and the ethics committees of the Institute of Tropical Medicine (ITM), Belgium (Approval number 864/13); the Antwerp University Hospital, Belgium (Approval number 13/7/85); and the University of Liverpool, UK (Approval number RETG000639IREC).

No sample size calculation was performed, because this was an exploratory laboratory sub-study on ring biofilm. Nevertheless, to answer the primary objective of the study, assessing the pre-post changes in the vaginal microbiome, a sample size calculation with 95% power to detect clinically important changes in bacterial counts defined 60 women in each group to be sufficient [16].

### Ring plus study

From 03 Jun 2013 until 19 Mar 2014, 120 adult female participants participated in the Ring Plus study. Participants used the NuvaRing^®^ contraceptive vaginal ring (Organon N.V., Oss, the Netherlands) over a period of three months [16]. The women had each ring inserted for three weeks continuously followed by one week off (intermittent use) or three weeks continuously with no breaks in between the removal of the old/insertion of the new ring (continuous use). Women in the intermittent use group used three rings each, and women in the continuous group each used four rings, during the whole study period. Vaginal examination, ring removal, and sample collection were carried out by the study clinician, as described previously [17]. For this sub-study, vaginal fluid was rolled on two microscopy slides and air-dried for each participant at baseline and at each ring removal visit. One slide was Gram stained for Nugent scoring, and the other slide was used to assess the presence of a vaginal biofilm.

All rings worn by study participants were collected. Each ring was cut in three equal parts immediately after removal. The part for the biomass density assessment with crystal violet was submerged in 3 ml of glutaraldehyde for two weeks, transferred to 3 ml of formaldehyde, and stored at 2–8°C until testing. This part was also used for scanning electron microscopy (SEM) after the crystal violet assay. The part for quantitative polymerase chain reaction (qPCR) was stored in diluted phosphate buffered saline (dPBS) (pH 7.4–1:9, PBS:saline) at -20°C. The final part for fluorescence microscopy was stored in Carnoy solution (6:3:1, ethanol:chloroform:glacial acetic acid) at 2–8°C until testing [14]. All samples, except for the Nugent slides, were shipped from the study site in Rwanda to the Institute of Tropical Medicine (ITM) in Antwerp, Belgium: the refrigerated samples were transported at room temperature, while the frozen samples were transported at -196°C in a dry shipper.

### Laboratory assessment of clinical samples

The vaginal microbiota was characterised in two ways: Nugent scoring of Gram stained vaginal smears [18] in the on-site RU laboratory and confocal laser scanning microscopy (CSLM) after peptide nucleic acid (PNA) fluorescence in situ hybridisation (FISH) of a second vaginal smear to detect vaginal biofilm at the ITM in Antwerp. In Nugent scoring, a score of 0–3 is considered a normal vaginal microbiota; a score of 4–6 an intermediate microbiota and a score of 7–10 BV. Vaginal biofilms on another vaginal smear were visualised with CSLM after FISH. This technique was performed as described previously using three probes: the broad-range BacUni-1 probe detecting all bacteria, and probes detecting two bacterial species strongly associated with BV (AtoITM1 for *A*. *vaginae* and Gard162 for *G*. *vaginalis*) [14], [17].

The biomass on worn contraceptive rings was stained with crystal violet to determine the optical density (OD) as a proxy for the biomass quantity. The crystal violet microtiter plate assay [19] was adapted to fit the ring parts and applied to identify and measure the biomass density on the rings. First, the ring biomass was stained with crystal violet (0.1% solution) for 10 minutes. Next, the ring part was rinsed twice and air-dried and the ring biomass staining was solubilised by submerging the ring in 3 ml of 30% acetic acid in water. From this solution, 125 μl was transferred to a new microtiter plate for OD measurement at 550 nm. Bacterial biomass compositions were assessed by qPCR of the *Lactobacillus* genus, *G*. *vaginalis*, and *A*. *vaginae*. Frozen ring parts were thawed and vortexed; using this eluate, 200 μl DNA was extracted (Abbott, Maidenhead, UK) and stored at -80°C until testing. qPCR was performed for each bacteria genus or species separately. The PCR mixtures and primers for *A*. *vaginae*, *G*. *vaginalis*, and *Lactobacillus* genus and the amplification reactions (Rotor Gene Q MDx 5 plex, Qiagen, Venlo, the Netherlands) have been described before [14].

In a random sub-sample of 120 rings, bacterial compositions of the biomass were also visualised by CSLM after PNA FISH for *G*. *vaginalis*, *A*. *vaginae*, and *Lactobacillus* genus. Slides were prepared for fluorescence microscopy by rinsing the ring with ddH_2_O, removing the biomass attached to the ring and spreading it out on the microscopy slide, passing the slide through a flame twice, and fixating it in Carnoy solution. PNA FISH was performed as described earlier [17], [20]. An additional probe targeting the *Lactobacillus* genus (Lac663) [21] was used to visualise the *Lactobacillus* species in the biomass. Furthermore, we applied SEM on a random selection of 11 rings to enable a three-dimensional view of the biomass architecture. The ring parts were dehydrated in an ethanol line and critical point dried. The parts were cut in pieces of one centimetre each, mounted on metal specimen stubs, coated with a 16 nm thick platinum film, and imaged using a JEOL JSM-840 microscope.

### Microscopic evaluation

Fluorescence microscopy was conducted by one microscopist (LH) who recorded for each vaginal slide and ring part whether she visualised any bacterial biofilm (positive fluorescence signal for the “all bacteria” probe), a biofilm incorporating *G*. *vaginalis* and/or *A*. *vaginae* (positive fluorescence signals for the relevant species-specific probe), and/or whether she visualised any of these as dispersed/planktonic bacteria only. Biofilm was defined as a dense network of bacteria adhering to a surface (the vaginal epithelial cells), dispersed/planktonic bacteria were defined as scattered bacteria, not visibly adhering to other bacteria or a surface.

### Statistical analysis

STATA 14 was used to analyse data. The fluorescence microscopy results were presented in three ways. First, we noted whether any vaginal biofilm (“all bacteria” probe positive) was visualised, as well as biofilms containing *G*. *vaginalis* and/or *A*. *vaginae* (each of these was assessed for each slide and results are not mutually exclusive). Second, when no biofilm was visualised at all, we noted whether dispersed/planktonic bacteria were present (for all bacteria, *G*. *vaginalis*, and *A*. *vaginae*; not mutually exclusive). Third, when no biofilm and no dispersed/planktonic bacteria were visualised, we noted that no bacteria were visualised at all (for all bacteria, *G*. *vaginalis*, and *A*. *vaginae*; not mutually exclusive).

Ring biomass density was presented as mean OD with standard deviation for each comparison group of interest. For *Lactobacillus* genus, *G*. *vaginalis*, and *A*. *vaginae* in ring biomass eluates, the presence was presented as proportions and bacterial concentrations were presented in log_10_ transformed genome equivalents (geq)/ml, with 95% confidence intervals (CI) estimated by linear mixed effects models with random intercept and no covariates. We used simple linear mixed effects regression analysis with a random intercept for participant (due to repeated observations) to evaluate associations between ring biomass density and ring biomass species presence and concentrations, Nugent score categories, and fluorescence microscopy results (presence of *G*. *vaginalis* vaginal biofilm, *A*. *vaginae* biofilm, *G*. *vaginalis* in a dispersed form, and *A*. *vaginae* in a dispersed form).

## Results

The mean age of the 120 randomised participants was 28.4 years (95% CI: 25–32), with 61% of women being married, and 57.5% having attained more than just primary school education. All participants but one completed the study, which implies that 417 rings and matching vaginal smear duplicates (3 times 60, or 180 from the intermittent group; 4 times 60, or 237 from the continuous group, excluding 3 samples of one discontinued participant) should have been collected. We were able to collect a total of 415 vaginal rings and 415 vaginal smears in duplicate. Two sample sets did not reach the laboratories. A total of 415 vaginal rings were assessed for biomass with the crystal violet assay, 412 ring eluates by qPCR, and sub-samples of 120 rings by FISH and 11 rings with SEM. The quality of 7 slides was insufficient for Nugent scoring, leaving us with 408 vaginal slides to score, matching the 415 time-points for which a vaginal ring was available. FISH results were available for 362 vaginal slides matching the 415 time-points for which a vaginal ring was available. The quality of 53 samples was not sufficient for FISH.

### Vaginal microbiota

Most slides (n = 251, 61.5%) had a normal Nugent score of 0–3, 28.9% (n = 118) a BV Nugent score 7–10, and 9.6% (n = 39) an intermediate score of 4–6. A bacterial biofilm was present on 53% of vaginal slides, a *G*. *vaginalis* biofilm in 38.4% of samples, and an *A*. *vaginae* biofilm in 27.1% of slides (Table 1).

**Table 1:** Vaginal microbiome of participants at time of removal of contraceptive ring: presence and absence of a vaginal biofilm with confocal laser scanning microscopy after fluorescence in situ hybridisation by species. (53 results unavailable due to inadequate quality of samples for confocal laser scanning microscopy).

Fluorescence microscopy characteristic (n = 362)	n (%)
**Presence of vaginal biofilm**	
All bacteria	192 (53.0)
*Gardnerella vaginalis*	139 (38.4)
*Atopobium vaginae*	98 (27.1)
**Presence of dispersed species only**	
All bacteria	170 (47.0)
*Gardnerella vaginalis*	71 (19.6)
*Atopobium vaginae*	40 (11.0)
**Absence of species**	
All bacteria	0 (0)
*Gardnerella vaginalis*	152 (42.0)
*Atopobium vaginae*	224 (61.9)

### Presence, bacterial composition, and structure of the vaginal ring biomass

**Fig 1:**
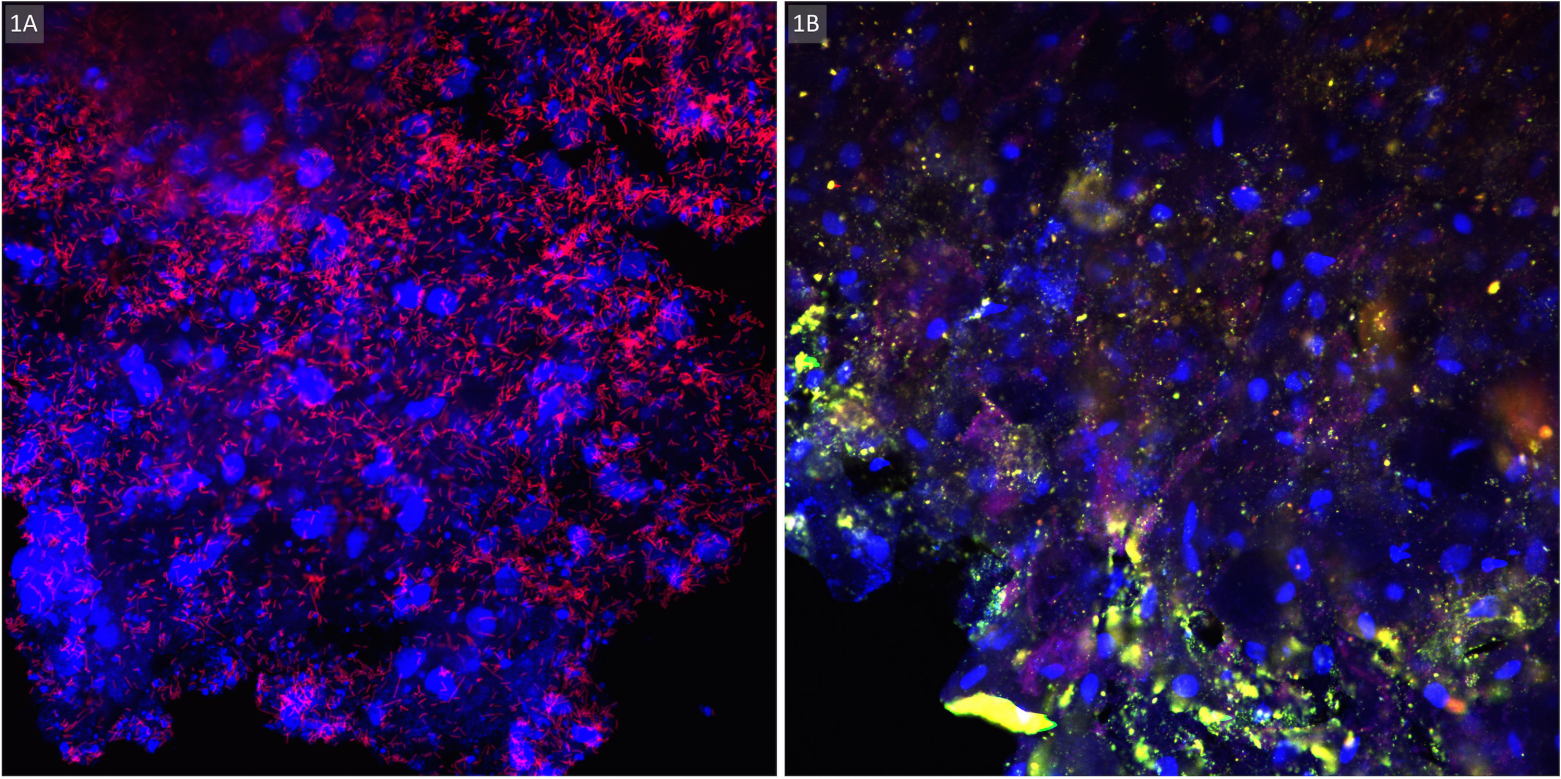
Visualisation of biomass on intravaginal ring surface by confocal laser scanning microscopy after fluorescence in situ hybridisation at 400x magnification: A. Lactobacilli (*Lactobacillus* spp. PNA-probe Lac663 with Alex Fluor 647 in red) scattered on vaginal epithelial cells (DNA stain with 4’,6-diamidino-2-phenylindole (DAPI) in blue); B. Vaginal epithelial cells DNA stain with DAPI in blue) partially covered with bacterial biofilm (*G*. *vaginalis* specific PNA-probe Gard162 with Alexa Fluor 647 in red and *A*. *vaginae* specific PNA-probe AtoITM1 with Alexa Fluor 488 in green).

Biomass was detected on all 415 rings using the crystal violet assay. The biomass OD ranged from 0.13 to 3.91 (mean OD 3.36; standard deviation (SD) 0.64). qPCR showed that the *Lactobacillus* genus was present in most ring eluates (93.2%, CI 89.9–96.5), with a mean log_10_ bacterial concentration of 6.20 (CI 6.07–6.33) geq/ml. *G*. *vaginalis* was detected in 237 eluates (57.4%, CI 50.4–64.4) mean concentration 5.99 (CI 5.80–6.17) geq/ml. *A*. *vaginae* was less common and quantified in 154 samples only (37.8%, CI 30.5–45.2; mean load 6.55, CI 6.27–6.84 geq/ml). Ninety ring biomass eluates contained *G*. *vaginalis* without *A*. *vaginae* being present whereas *A*. *vaginae* was only detected in seven ring eluates without *G*. *vaginalis*. The images of the fluorescence microscopy, on a subset of 120 ring biomass samples mounted on slides, showed a presence of lactobacilli in 77 (64.2%) of samples. *G*. *vaginalis* was seen in 74 (61.7%) and *A*. *vaginae* in 37 (30.8%) of the biomass samples mounted onto slides (Fig 1).

The presence of *A*. *vaginae* in the ring biomass eluate was associated (mixed effects modelling) with the biomass density (OD +0.18; 95% CI 0.05–0.32; p = 0.008) and showed a significant linear increase (OD +0.03; 95% CI 0.01–0.05; p = 0.002) for each log10 increase in *A*. *vaginae* concentration. The presence of *G*. *vaginalis* was not significantly associated with the ring biomass density (OD +0.10; 95% CI -0.03–0.23; p = 0.132), but the mean ring biomass density increased for each log_10_ increase in *G*. *vaginalis* concentration (OD +0.03; 95% CI 0.01–0.05; p = 0.013). For the *Lactobacillus* genus, neither the presence (OD -0.03; 95% CI -0.28–0.22; p = 0.816), nor the concentration (OD -0.00; 95% CI -0.04–0.03; p = 0.991) was significantly correlated with the ring biomass density (Table 2).

**Table 2:** Association of the vaginal microbial status with contraceptive vaginal ring biomass.

Risk factor	Mean change in density for a one unit change in risk factor	95% confidence interval	p-value from regression analysis*
**Diagnosis bacterial vaginosis**			0.002
Normal Nugent score (0–3)	Ref		
Intermediate Nugent score (4–6)	+0.09	-0.12–0.30	
Bacterial vaginosis Nugent score (7–10)	+0.26	0.11–0.41	
**Fluorescence microscopy after FISH by species**			
*Gardnerella vaginalis* biofilm	+0.24	0.10–0.38	0.001
*Gardnerella vaginalis* dispersed	+0.10	-0.04–0.24	0.147
*Atopobium vaginae* biofilm	+0.22	0.06–0.37	0.005
*Atopobium vaginae* dispersed	+0.09	-0.05–0.24	0.195
**Quantitative polymerase chain reaction**			
*G*. *vaginalis* present in ring biomass	+0.10	-0.03–0.23	0.132
For each log_10_ increase of *G*. *vaginalis* in ring biomass	+0.03	0.01–0.05	0.013
*A*. *vaginae* present in ring biomass	+0.18	0.05–0.32	0.008
For each log_10_ increase of *A*. *vaginae* in ring biomass	+0.03	0.01–0.05	0.002
*Lactobacillus* spp. present in ring biomass	-0.03	-0.28–0.22	0.816
For each log_10_ increase of *Lactobacillus* spp. in ring biomass	-0.00	-0.04–0.03	0.991

*Mixed effect regression analysis corrected for participant multiple observations (random intercept).

**Fig 2:**
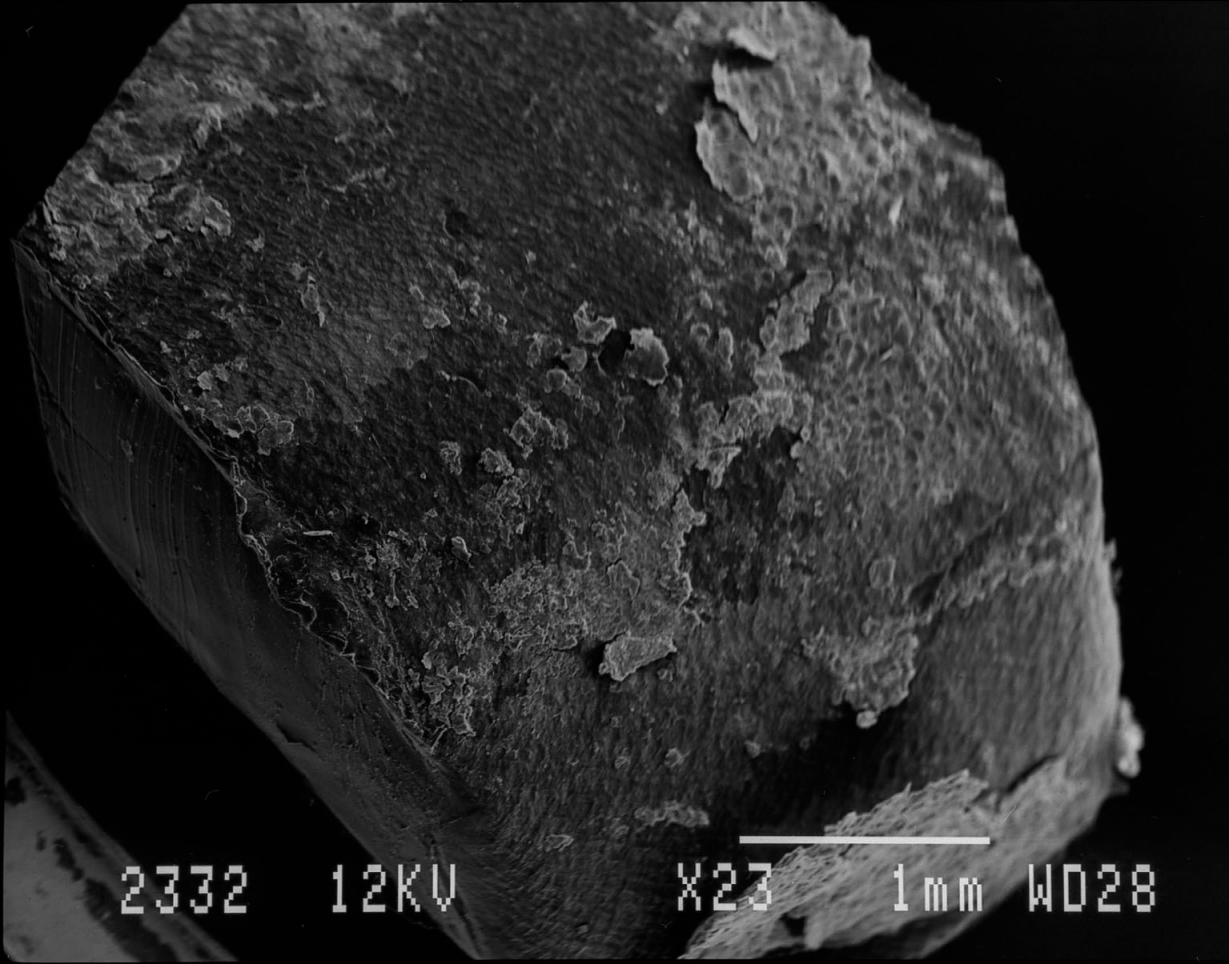
Visualisation of biomass on intravaginal ring surface by scanning electron microscopy at 23x magnification.

**Fig 3:**
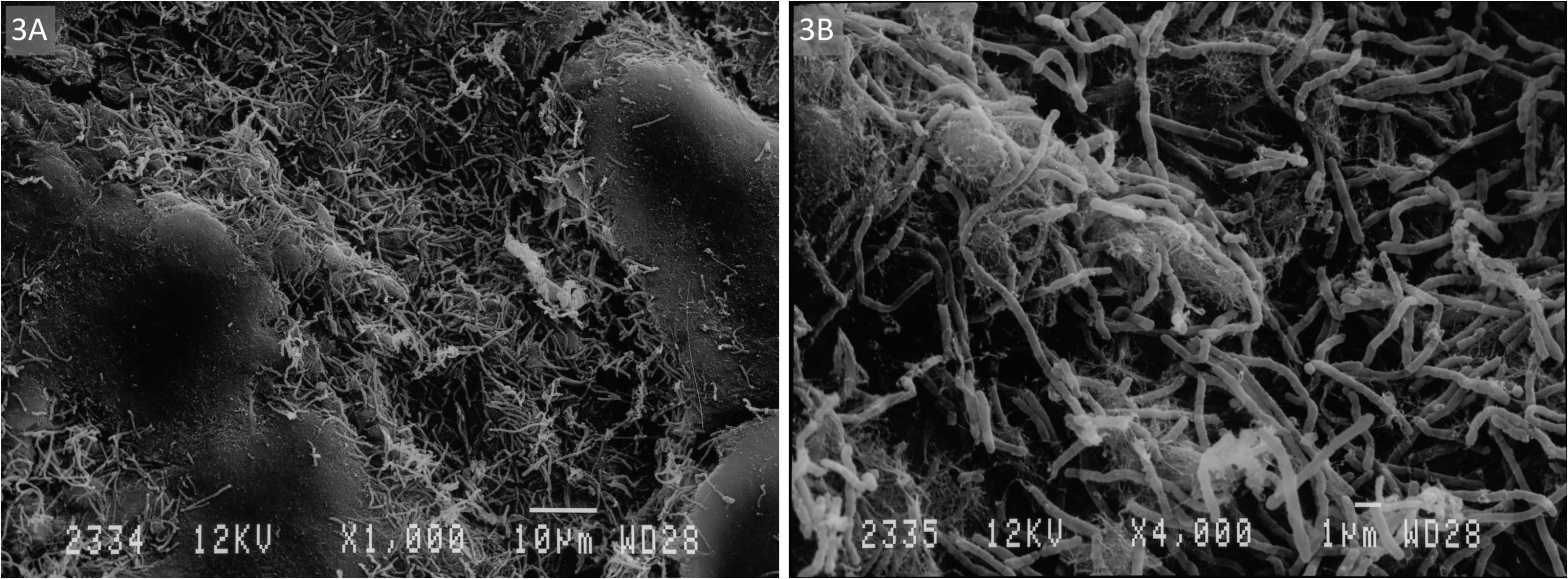
Visualisation of biomass on intravaginal ring surface by scanning electron microscopy at A. 1000x and B. 4000x magnification: Phenotype 1—elongated bacteria scattered on vaginal epithelial cells.

**Fig 4:**
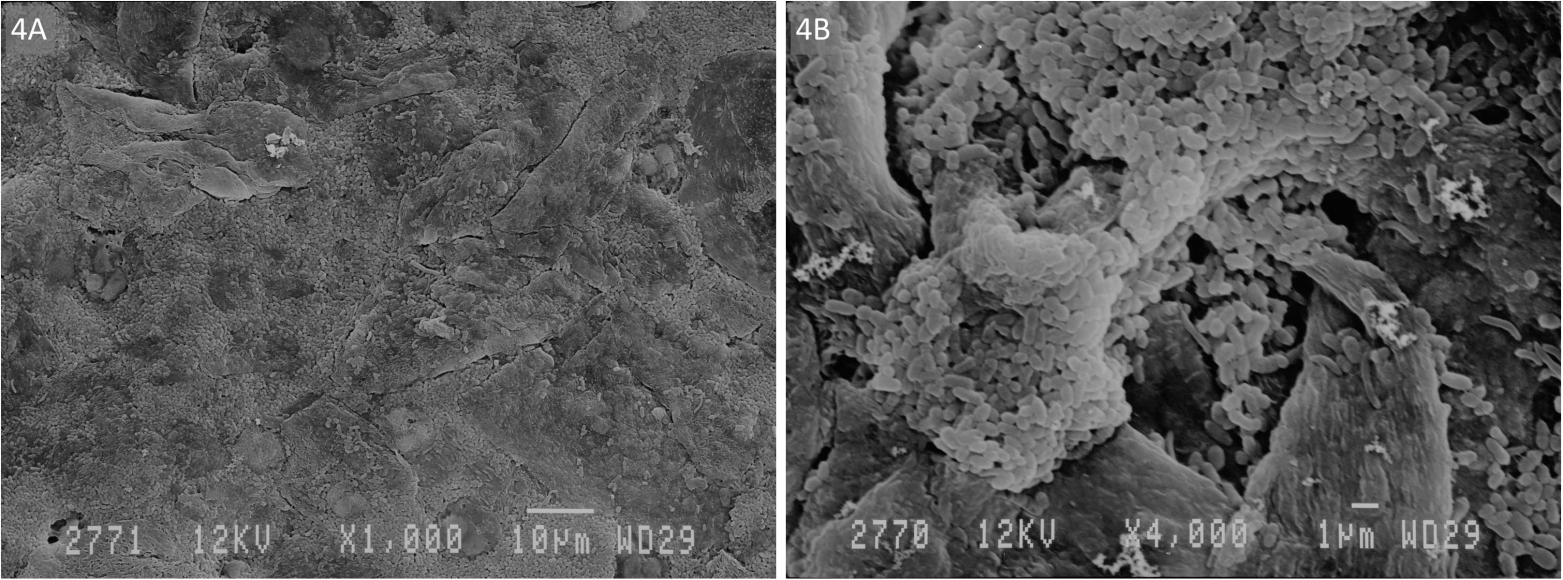
Visualisation of biomass on intravaginal ring surface by scanning electron microscopy at A. 1000x and B. 4000x magnification: Phenotype 2—condense biofilm of bacilli on vaginal epithelial cells.

SEM on a subset of 11 rings showed that all rings were covered with layers of vaginal epithelial cells and bacteria with diverse shapes and sizes adhered to these epithelial cells (Fig 2). We differentiated two phenotypes (Figs 3 and 4). The first type consisted of a loose network of scattered elongated bacteria. The second type was characterised by a dense bacterial biofilm with bacilli. All seven rings categorised in the first phenotype had matching vaginal samples that were scored as Nugent 0–3 (n = 6) or 4–6 (n = 1), while the three rings with phenotype 2 had matching vaginal samples scored as Nugent 8–10.

### Association of the vaginal microbiota compositions and the ring biomass density

Mean ring biomass densities were compared among the three Gram stain Nugent score categories (for rings and Gram stain slides that were collected together: from the same participant at the same study visit). Mixed effect regression analysis showed that vaginal ring biomass in the BV Nugent score category (OD +0.26; 95% CI 0.11–0.31) and intermediate score category (mean OD +0.09; 95% CI -0.12–0.31) had a statistically significantly higher mean density compared to ring biomass in the normal score category (p = 0.002 (Table 2). The presence of a vaginal biofilm containing either *G*. *vaginalis* and/or *A*. *vaginae* by FISH fluorescence microscopy also correlated with a higher ring biomass density (mean OD +0.24; 95% CI 0.10–0.38; p = 0.001 and mean OD +0.22; 95% CI 0.07–0.37; p = 0.005 respectively); Table 2). No significant associations between the presence of planktonic/dispersed vaginal *G*. *vaginalis* and *A*. *vaginae* and ring biomass density were found.

## Discussion

This laboratory sub-study of a vaginal contraceptive ring trial in African women showed that the formation of biomass on the vaginal rings that had been worn for three weeks was common and present in varying densities. We demonstrated that lactobacilli were nearly always part of the ring biomass and that bacteria playing an important role in BV were often present: *G*. *vaginalis* in more than half of the ring biomasses and *A*. *vaginae* in more than one-third. The concentrations of these two bacteria in ring eluates were positively associated with ring biomass density, indicating that a denser biomass likely consists of higher numbers of the bacteria. In addition, we showed that vaginal microbiota dysbiosis (defined as a Nugent score of 7–10 and 4–6) or vaginal biofilm presence (visualised by fluorescence microscopy) were associated with a higher mean ring biomass density. These findings suggest that the status of the vaginal microbiota influences the formation or deposit of biomass on vaginal rings and/or vice versa. Our study was cross-sectional and therefore does not allow us to determine temporality and causality of these associations.

Only two other human studies and one macaque study have visualised the surfaces of vaginal rings after use. Miller et al. applied electron microscopy to examine a NuvaRing used for four weeks by a healthy volunteer, and observed cellular debris but no bacterial growth on the surface of the ring [22]. We speculated that the magnification of 200X that they used was too low to visualise bacteria. In comparison, we used magnifications of 1000X and 4000X in the present study. A second study in human volunteers showed the presence of biomass on all 48 rings containing an antiretroviral drug that were used for four weeks [23]. SEM with a magnification of 25X was used to semi-quantify the biomass density. In this population of women, of whom more than two-third had a normal Nugent score, the ring biomass density (semi-quantified visually with SEM) was not associated with the Nugent score category [23]. Gunawardana et al. [24] differentiated two biomass phenotypes, while visualising the surface of vaginal rings worn by six female pig-tailed macaques for 28 days with electron microscopy and fluorescence microscopy. They found large areas of the ring surface covered with tightly packed mats of bacteria and epithelial cells or thicker interwoven networks of uniform fibres. We also differentiated two phenotypes. The first type consisted of a loose network of scattered elongated bacteria, probably lactobacilli, which agrees with the normal Nugent score of the matching vaginal smears. The second type was characterised by a dense bacterial biofilm with bacilli, also in agreement with the BV Nugent score of the associated vaginal smear.

At present, contraceptive vaginal rings are commonly used in countries where HIV is not endemic and BV prevalence is low. However, multipurpose and long-acting vaginal rings for the prevention of HIV and pregnancy are being developed specifically for use in HIV-endemic countries, most of which are in sub-Saharan Africa [3], [9], [25–28]. Recently, a vaginal ring containing the antiretroviral drug dapivirine was shown to be effective for HIV prevention in sub-Saharan African women [2]. It has to be noted that BV prevalence in sub-Saharan Africa is high [29], [30]. Furthermore, as demonstrated in the current study, the presence of vaginal dysbiosis and BV-associated bacterial biofilm is associated with a denser biomass on used vaginal rings. The effect of this biomass should be investigated in order to warrant safe and effective use of intravaginal rings. Extensive epidemiological research has shown that sex hormones, including those released by contraceptive vaginal rings, have a beneficial effect on the vaginal microbiome [8–10], [31]. Incorporating oestrogen and/or progestogens in vaginal rings may therefore be an important strategy to protect the vaginal microbiota during ring use in addition to protecting against pregnancy. Other components that are beneficial for the vaginal microbiome, such as acidifying agents and probiotic lactobacilli, could also be added to vaginal rings in the future.

We have previously shown that the ability of bacteria to adhere to the device surface differs by the type of material used [32]. We showed that adherence of gonococci was greater on the silicone ring material as compared to the thermoplastic ring material [32]. The NuvaRing is composed of a thermoplastic (ethylene-vinyl acetate copolymer) material, but it is unclear if BV-associated bacteria would behave similarly to gonococci in vitro, and if in vitro data accurately predict what would happen in vivo.

In summary, our study showed that biomass easily forms on the contraceptive vaginal ring within three weeks and that BV-associated bacteria are commonly present in this biomass. Our study also showed associations between the presence of vaginal dysbiosis and vaginal biofilm and the ring biomass density. The temporality and causality of these relationships deserve further study. Furthermore, we recommend that the design and development of multipurpose vaginal rings take ring biomass formation into account by studying the effects on the vaginal microbiota and active pharmaceutical ingredient release.
